# Bio-Fibres as a Reinforcement of Gypsum Composites

**DOI:** 10.3390/ma14174830

**Published:** 2021-08-25

**Authors:** Alessandro P. Fantilli, Daria Jóźwiak-Niedźwiedzka, Piotr Denis

**Affiliations:** 1 Department of Structural, Geotechnical and Building Engineering, Politecnico di Torino, Corso Duca degli Abruzzi 24, 10129 Torino, Italy; 2 Institute of Fundamental Technological Research Polish Academy of Sciences, Pawińskiego 5b, 02-106 Warsaw, Poland; djozwiak@ippt.pan.pl (D.J.-N.); pdenis@ippt.pan.pl (P.D.)

**Keywords:** organic waste material, fibre-reinforced gypsum, mechanical properties, microstructure

## Abstract

Three series of tests performed on fibre-reinforced gypsum composites are described herein. Sheep wool fibres and hemp fibres were used as reinforcement. The aim was to evaluate the capability of these biomaterials to enhance the fracture toughness of the gypsum matrix. The mechanical properties were measured by means of flexural tests on small specimens, whereas scanning electron microscopy with energy dispersive spectroscopy and X-ray diffraction were used to analyse the microstructure and composition of the fibres and of the gypsum composites. As a result, wool fibres were shown to improve the mechanical performance of the gypsum matrix, better than hemp fibres. This is due to the high adhesion at the interface of the fibre and gypsum matrix, because the latter tends to roughen the surface of the wool and, consequently to increase the bond strength. This preliminary research carried out shows that this type of biofiber—a waste material—can be considered a promising building material in sustainable and environmentally friendly engineering.

## 1. Introduction

In the construction sector, industrial fibres made with glass, basalt, polymer (polypropylene, polyester or PVA) or steel, play a fundamental role in improving the behaviour of brittle matrix composites. Under tensile loads, a fibre-reinforcement can increase the fracture toughness and the resistance to crack propagation. The tensile strength of the brittle composites also improves, especially when a large volume of fibres is added to the matrix [1]. The application of various fibre-reinforced composites is currently growing, driven by many industrial branches. New fibres, with high performance and low production cost, can improve existing composites and enlarge their possible applications. Apart from the common man-made fibres, the interest in organic fibres has recently increased. As animal and vegetal fibres are eco-friendly materials, annually renewable, and totally recyclable, they meet the requirements of green building rating systems and therefore they are frequently used as construction materials [1].

The European sheep population is composed of 80 million animals [2], and each of them produce about 1.5–3 kg of wool per year [3]. For several reasons, e.g., lower quality than the Merino wool produced in Australia and New Zealand, about 200 million tons of sheep wool are currently considered as special waste and must be disposed of. Wool can be a valuable resource also for the construction industry, because it is an eco-friendly material and meets the requirements of green building rating systems [1]. Moreover, in the production of new building components, wool can be used without any chemical treatment. This is the case for some cement-based composites, whose thermal and acoustic insulations increase with the content of wool [4,5,6].

The addition of sheep wool fibres, as well as hemp fibres, treated or not with atmospheric plasma, can also improve the mechanical performances of cementitious mortars [7,8]. In this context, bio-fibres can reinforce the cement-based composites, similar to industrially manufactured polymeric fibres. However, the practical application of natural fibres as reinforcement must be supported by studies concerning their chemical degradation in alkaline environment, like that produced by cementitious systems [9]. 

Alternatively, animal and vegetal fibres can be used to reinforce brittle matrixes made with gypsum, which is widely applied as a construction material [10,11]. As is known, gypsum-based composites are also used to finish surfaces of new and renovated walls, and ceilings. Partition elements made with gypsum-based plaster also act as an acoustic and fire barrier. The brittleness of these manufacts may be appreciably reduced by reinforcing gypsum with natural fibres [12], instead of using polymeric [13,14] and glass [15] fibres. As demonstrated in some research works [16,17], the hemp reinforcement of gypsum plaster is both cost-effective and environmentally friendly. 

Recently, Jia et al. [18] listed over 10 types of natural fibres capable of reinforcing gypsum-based composites, without taking into account animal fibres, including sheep wool fibres. For these reasons, the feasibility of reinforcing gypsum composites using wool, as an alternative to other bio-fibres, is investigated herein for the first time. In addition, all the existing studies mainly focus on the macroscopic properties of materials, without investigating the microscopic properties, such as the adhesion of fibres with the matrix. As sheep wool, which is a waste material, provides the same performances of hemp in fibre-reinforced cement-based mortars [7], the goal of this work was to analyse the feasibility of reinforcing gypsum composites using wool as a valid alternative to vegetable fibres. A detailed microstructure analysis was also performed to investigate the adhesion of animal and plant origin fibres in gypsum-based composites, which is the key factor influencing the reinforcement effect. 

The aim of the research was to introduce a new application in which wool waste can be easily implemented and valorised without adopting any chemical treatment. Thus, in all the sections of the present paper a comparative analysis between hemp and wool fibres is performed. In particular, Section 2 establishes the experimental framework for deriving the effective properties of the materials, whereas Section 3 recalls the procedure for measuring the mechanical performances and the microstructure analysis of gypsum composites. The results of the tests carried out on plain and fibre-reinforced gypsum samples are finally presented and discussed in Section 4.

## 2. Materials and Methods

### 2.1. Constituent Materials

**Figure 1:**
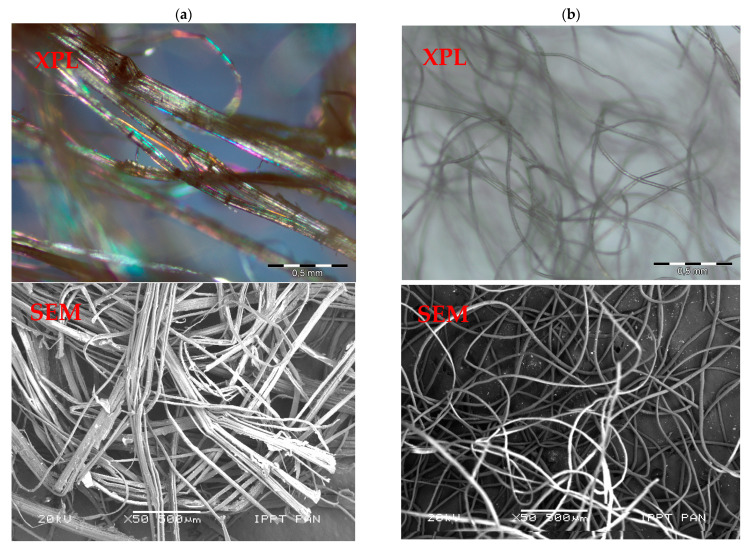
Microphotographs of fibres in transmitted cross polarized light (XPL) and in a scanning electron microscope (SEM): (**a**) hemp, and (**b**) sheep wool; scale bar = 500 µm.

**Figure 2:**
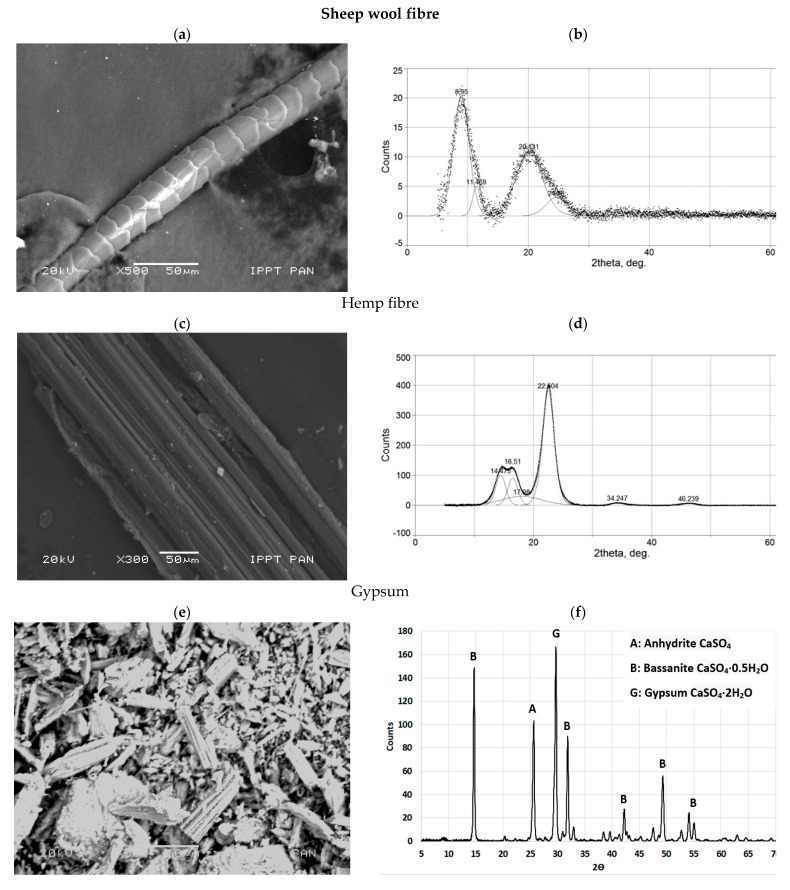
SEM microstructure and X−ray diffraction (XRD) diffractograms of (**a**,**b**) sheep wool fibre, (**c**,**d**) hemp fibre, and (**e**,**f**) gypsum.

Two different natural fibres were considered: animal origin-sheep wool fibres and plant origin-hemp fibres. Raw wool fibres contain a number of constituents other than the fibre, such as grease, water-soluble material derived from perspiration, and contaminants [1]. However, the sheep wool investigated herein was previously washed to remove all impurities, like dirt and grease. Wool fibres have a density of 1.0 g/cm^3^ and an average diameter of 20 µm, whereas the diameter of the hemp fibres (having the same density of wool) ranges from 10 µm to 250 µm. The reason for such variation can be seen in Figure 1, where hemp fibres with a large diameter tend to delaminate into fibres of small diameters (see Figure 1a) even in the absence of external actions, whereas wool filaments (see Figure 1b) do not disaggregate. The detailed microstructures of sheep wool, hemp fibres and gypsum, including the chemical composition, were determined using scanning electron microscopy (SEM), as shown in Figure 2a,c,e.

The main crystalline compounds detected by X-ray diffraction (XRD) are shown in Figure 2b,d,f. Sheep wool fibres (Figure 2a) consist of α-helix (peaks at around 9° and 11.5°) and β-sheet structures (peaks at around 20.1° and 24.5°). In the literature, they are described as crystal type structures but with a relatively low degree of crystallographic order formed from random coil structures present in keratin [19].

As shown in Figure 2b, the crystal peaks relating to hemp fibres (with one amorphous peak at 17.98°) can be due to a higher order of crystallographic structure than that present in wool fibres. It has already been called a semi-crystalline structure, with a relatively high degree of crystallinity (84.5% calculated by PeakFit deconvolution). Comparing wool and hemp, it can be assumed that the ordered structure is mainly represented by an α-helix crystal peak (at around 9°) in wool, whereas in hemp the structure is mainly represented by a strong crystal peak at around 22.5°.

The differences in the XRD peak positions correspond to completely different d-spacings between repeating elements of the ordered structures. About three times shorter d-spacings for hemp may result in a much stiffer material, because of the more densely packed structure, showing a higher tensile strength. However, if they are used to reinforce a matrix, other aspects, like the filling material-matrix effects and the adhesion between components, have to be taken into consideration. The SEM observation of hemp fibres (Figure 1a and Figure 2c) reveals the presence of impurities which were mainly lignin, wax, and pectin. A special hemp treatment could modify the surface properties and would improve the bond between the fibres and the matrix. However, not all the treatments can have beneficial effects. Indeed, Ghoson et al. [20] found that some impurities were still present on the fibre surface even after silane treatment. 

The X-ray diffractogram of hemp fibres (Figure 2d) reveals a major crystalline peak, which occurs around 2*θ* ≈ 22.5°, and corresponds to the crystallographic plane of cellulose. Ghosn et al. [20] observed that the intensity of the XRD major peak in untreated and treated hemp fibres is different and indicates a variation in the crystallinity of the fibres. The crystallinity index *(I_c_*) can be calculated according to the following equation:

(1) $$I_{c}=\left(\frac{I_{002}-I_{am}}{I_{002}}\right)\times 100$$

where, *I_002_* = the maximum intensity of diffraction of the peak at a 2*θ* angle of 22.5°, and *I_am_* = the intensity of diffraction of the amorphous material at a 2*θ* angle of 19°. By using Equation (1), Ghosn et al. [20] found that alkali and acetyl treatments increase the intensity of the main peak while silane treatment tends to decrease it. However, in the hemp fibres used herein, the crystallinity index *I*_c_ was 84.5%, which is higher than the values measured by Ghosn et al. [20] (i.e., *I*_c_ lower than 73.20%). 

SEM analysis of gypsum particles showed various crystals, without any predominance of euhedral crystals, Figure 2c. The formation of prismatic euhedral crystals with sharp edges is important to obtain the best performance in terms of the compressive strength of the paste, providing more links between the hydrated products and acting as nucleation points [21]. The main minerals identified in the gypsum are as follows: gypsum (CaSO_4_·2H_2_O), bassanite (CaSO_4_·0.5H_2_O), and anhydrite (CaSO_4_) (see Figure 2c). 

Sheep wool fibres mainly consisted of carbon (70%), sulphur (15%), nitrogen (6%), and traces of calcium (2%), while hemp fibres were characterized by a higher content of carbon (67%) and traces of calcium (1.5%), silica (0.95), sulphur (0.8%), and magnesium (0.3%). Gypsum consisted mainly of calcium (40%) and sulphur (30%) with traces of carbon (4%), silica (1%), and magnesium (0.5%).

### 2.2. Fibre-Reinforced Gypsum Composites

Sheep wool and hemp fibres have been already used in research on cement-based composites reinforced by natural bio-fibres [7]. A similar experimental approach is used herein to investigate, for the first time, gypsum-based composites, with and without sheep wool fibres, and compare them with those reinforced by hemp fibres. 

Three series of gypsum-based composites were made: ref. without fibres, WF with sheep wool fibres, and HF with hemp fibres. The composite of Series ref. was only composed of plain gypsum paste, with a water-binder ratio w/b = 0.47. Wool fibres were added to Series WF in an amount of 12 g (about 1% in volume), whereas the w/b of the gypsum paste increased (w/b = 0.49), because of the water absorption of the fibres. In addition, in the Series WF, reinforced with 12 g of hemp (about 1% in volume), the gypsum paste had a w/b = 0.52, larger than those of the other series due to the water absorbed by the fibres. In both the WF and HF series, 1% of in volume of fibres was the maximum reinforcement that could be added without compromising the workability of the final composites.

Three prisms (40 × 40 × 160 mm^3^) with each series were cast. All the prisms were cured in normal laboratory conditions (20 ± 1 °C with 50 ± 5% RH) and, after 75 days, the specimens were tested in bending compressions. Afterwards, detailed microstructure analysis was carried out on all the gypsum-based composites.

### 2.3. Mechanical Tests

**Figure 3:**
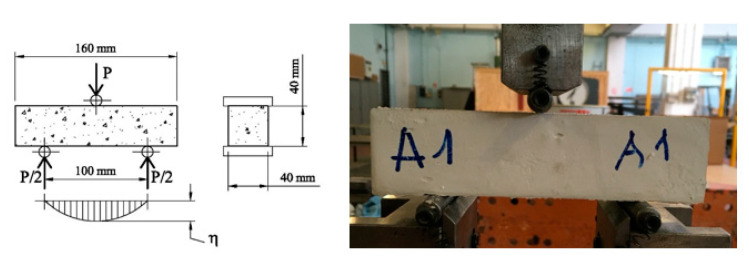
Three-point bending test on gypsum composites.

**Figure 4:**
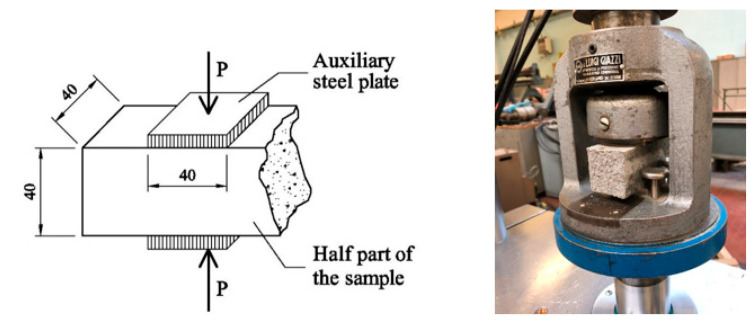
Compression test on gypsum composites.

According to EN 196-1 [22], the flexural strength can be measured by means of the three-point bending test. A testing machine, having a loading cell of 10 kN, was used. The apparatus is provided with two steel supporting rollers spaced 100 mm and (having a diameter of 10 mm). A third steel roller, of the same diameter and placed centrally between the other two, is used to apply the load P, Figure 3. In particular, the test was performed by driving the displacement of this roller, which was moved at a velocity of 0.05 mm/min. The same testing machine, but with a different apparatus, was also used to determine the compressive strength. The apparatus consists of a pacing device to facilitate the control of the loading, and of two auxiliary steel plates, having a thickness of 10 mm and a loading area of 40 × 40 mm^2^. The compressive load P is smoothly increased at the rate of 200 N per second until failure. The compression test was performed on one of the two halves of the specimen broken during the flexural test, Figure 4.

### 2.4. Microstructure Analyses of Gypsum Composites

X-ray diffraction was used to estimate the main minerals in the investigated components of the gypsum-based composites, as well as the crystal structures of the gypsum paste within the specimens. After drying at 105 °C, the gypsum specimens were powdered and sieved through a 0.045 mm sieve. The bio-based fibres were dried ar 105 °C and densely packed in a special container designed for XRD testing. XRD diffractograms were obtained at room temperature with a Bruker D8 DISCOVER diffractometer (Cu Kα radiation source) equipped with a Göbel mirror and a GADDS 2D detector system (Karlsruhe, Germany). The operation parameters of the equipment are 40 kV and 40 mA. The diffraction patterns were collected over a 2θ range from 5° to 70° with a 1°/min step by flat plane geometry. The Diffrac.Eva V5.2 software was used to evaluate the XRD patterns and to determine the crystallite phases. The size, texture, and mutual packing of the fibres were observed by means of an optical microscope BX51 in transmitted cross polarized light (XPL).

The morphological characteristic of pastes was investigated on a fresh split surface using a JEOL JSM-6460 LV high vacuum scanning electron microscope (Freising, Germany) with an energy dispersive spectroscopy (EDS) analyser and a resolution of 3.0 nm. Split specimens were sputtered with carbon (~20 nm) in a Cressington C208 sputtering device to make the surface electrically conductive, to prevent charging under the electron beam. A carbon conductive tap was also applied. A voltage of 15 kV and an aperture of 120 µm were used, whereas the working distance was 11 mm. The observations were made using a magnification range of 30× to 1500×. The chemical composition of the specimens was determined using standardless analytical algorithms, where the element concentrations were normalized to 100%. EDS spectra were collected from more than 20 different zones (of 1 × 1 µm^2^) on each homogeneous specimen. The list of the analysed elements was created automatically on the basis of all the peaks, as identified by the analytical software Genesis Spectrum 6.2 by EDAX Inc and confirmed by the operator.

## 3. Test Results and Discussion

### 3.1. Mechanical Performances

**Figure 5:**
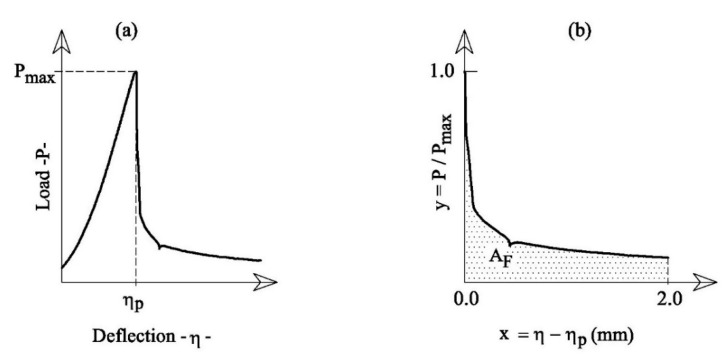
The results of the flexural tests: (**a**) deflection-load and (**b**) post-peak curves.

Figure 5a shows the typical deflection–load curves, η-*P*, measured during the flexural tests. The ascending branch of this curve finishes at the maximum load *P_max_*, in correspondence to which the flexural strength of the plaster, *σ_flex_*, can be computed. More specifically, under the hypothesis of linear elastic behaviour of materials and assuming that the plane section remains plane, the following formula can be used:

(2) $$\sigma_{flex}=\frac{3}{2}\frac{P_{max}\;L}{B\;H^{2}}$$

where B = H = 40 mm = width and depth of the cross-section, and L = 100 mm = span of the beam. 

The descending branch of the η-*P* curve defines the ductility of the gypsum-based composites. In accordance with the tests already performed on cement-based wool-reinforced mortars (Fantilli et al., 2017), the flexural toughness can be defined by means of the anelastic displacement, x= η-η_p_, vs. the non-dimensional load, y= P/*P_max_*, as reported in Figure 5b. The area A_F_ delimited by the curve x-y (Figure 5b), up to x = 2 mm, was used herein to quantify the flexural toughness. 

**Figure 6:**
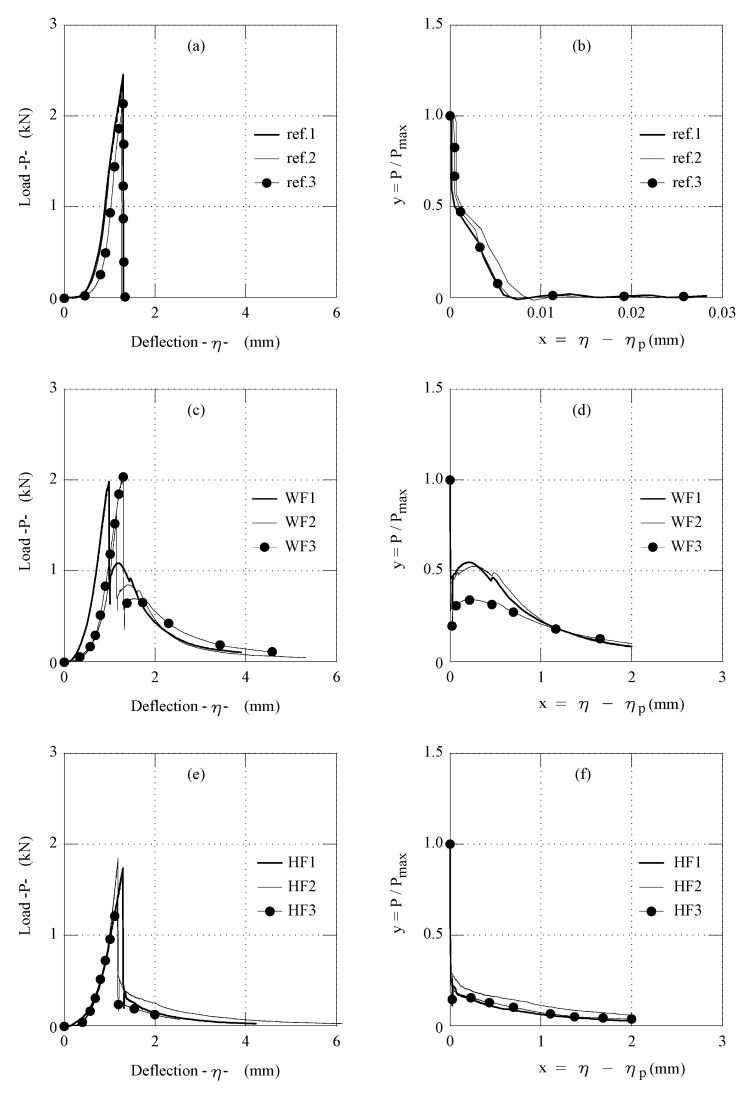
η-*P* and x-y curves experimentally obtained with the flexural tests; reference specimens A—(**a**,**b**), specimens with sheep wool fibres B—(**c**,**d**), specimens with hemp fibres C—(**e**,**f**).

All the η-*P* curves obtained from all the flexural tests performed on the three series of the gypsum composites are illustrated in Figure 6. In the same figure, the post-peak diagrams are also illustrated in terms of x-y. Finally, the main flexural properties, (i.e., *P_max_*, *η_p_*, and *A_F_*) are collected in Table 1. In the last column of this Table, the values of the maximum compressive load, *P_c_*, are also reported. 

From the values of *P_c_*, the compressive strength, *σ_c_*, of the gypsum composites can be calculated with the following formula:

(3) $$\sigma_{c}=\frac{P_{c}}{B\;H}$$

where B = H = 40 mm = the size of the auxiliary plates, on which *P_c_* is applied.

**Figure 7:**
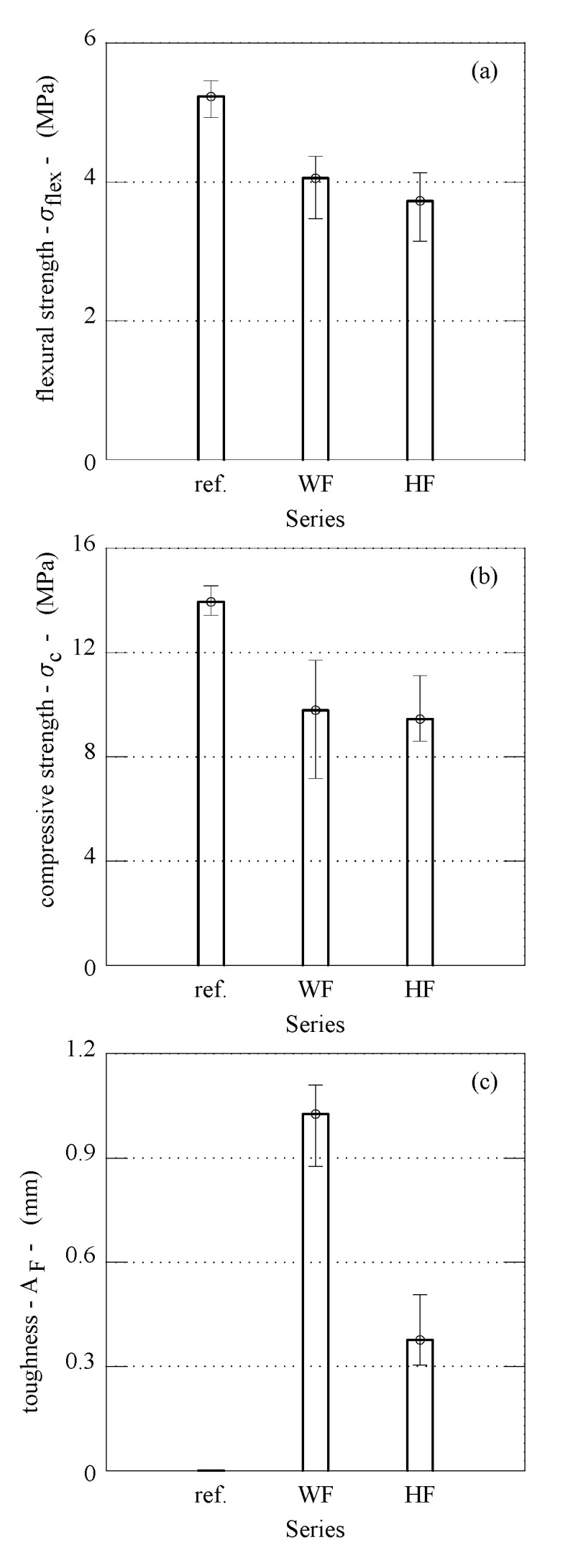
Average values of: (**a**) flexural strength, (**b**) compressive strength, and (**c**) flexural toughness of gypsum composites.

To compare the performances of all the gypsum-based composites investigated herein, the average values of *σ_flex_* and *σ_c_* are reported in the histograms of Figure 7a,b, respectively. In both wool (Series WF) and hemp (Series HF) fibre-reinforced gypsum, the compressive and flexural strengths are 20% lower than those of plain gypsum (Series ref.). This is due to both the higher water/binder ratio and, according to literature data [14], to the large content of fibre (1% in volume) as well.

Vice-versa, the average flexural toughness, which indicates the capability of the composite to maintain residual stresses on crack surfaces, is practically zero in the absence of fibres. This is evident in Figure 7c, where the average values of A_F_ are compared in the three series. However, in this histogram, the flexural toughness of Series WF, reinforced with wool fibres, is about 2.7 times that of the hemp-reinforced gypsum (Series HF). 

These results are consistent with those obtained by reinforcing gypsum composites with industrial fibres. Zhu et al. [14] reinforced gypsum paste (w/b = 0.6) with 1.2% in volume of polypropylene fibres having a diameter of 10 μm and a length of 6 mm. This gypsum composite showed a flexural strength of 4.84 MPa (similar to the flexural strength of Serie WF), and a toughness in bending *A_F_* = 0.64 mm which is only 60% of that obtained by using wool fibres. According to Mukhametrakhimov et al. [13], who investigated the influence of the type and length of reinforcing polypropylene fibre in gypsum-based composites, higher flexural strength and toughness can be achieved by increasing the length and volume content of the fibres. However, large volumes of polymeric fibres lead to their clumping and, consequently, mechanical performances decrease. Iucolano et al. [12,17] also found that the flexural strength is more or less the same, when 1% in volume of hemp fibres or glass fibres are used to reinforce the gypsum paste (w/b = 0.7), but A_F_ generated glass fibres are 1.5 times larger than those provided by hemp fibre. In both WF and HF composites, the scatter of *A_F_* is not large (see Table 1 and Figure 7c), which means that a good dispersion of fibres was achieved in the two composites, despite only 1% in volume of fibres being added. This result, compared to the values of A_F_ reported in Figure 7c, shows that wool fibres can bridge the cracks better than glass fibres. In other words, wool, which is nowadays a special waste, is a valid substitute of hemp, as well as of the current manufactured fibres used to reinforce gypsum.

### 3.2. Microstructure Analysis

**Figure 8:**
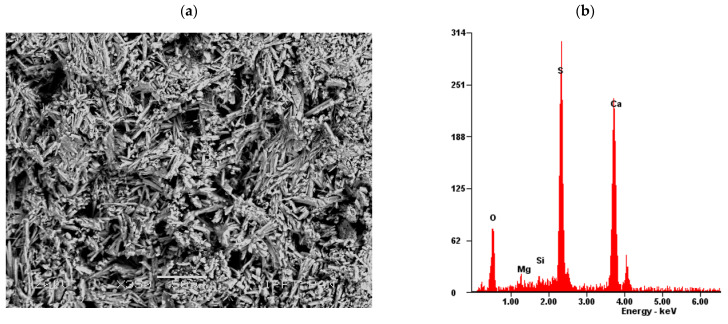
The analysed fresh split surface of gypsum paste: (**a**) SEM microphotograph and (**b**) EDS analysis in the reference specimen without fibres—Series ref; S-Sulphur, Ca-Calcium, Mg-Magnesium, Si-Silicon (scanning electron microscope, scale bar = 50 µm).

A detailed SEM-EDS analysis on the fresh split gypsum-based paste surface shows some differences in the microstructure due to the presence of different fibres. The reference gypsum paste without the addition of fibres (Series ref.) is characterized by a uniform microstructure. There are visible pillars of gypsum crystals of similar composition and size, as shown in Figure 8. The gypsum crystals are also characterized by basically hexagonal faces and rod-like shapes.

**Figure 9:**
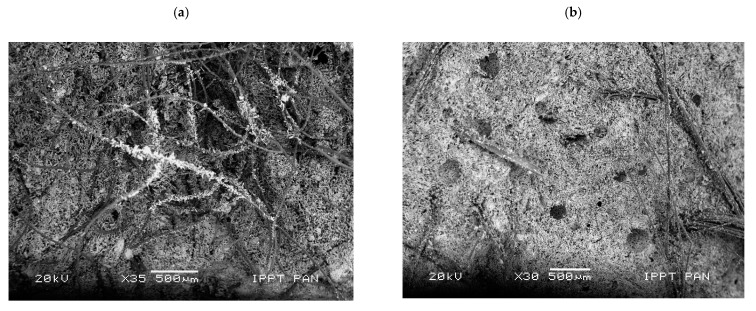
Microphotograph of the analysed fresh split surface of gypsum-based composites: (**a**) with sheep wool fibres and (**b**) with hemp fibres (scanning electron microscope, scale bar = 500 µm).

In the other series, the microstructure is directly influenced by the presence of fibres. Spherical empty air-voids are visible in the paste with hemp fibres (Figure 9b), whereas the gypsum matrix with sheep wool was characterized by a dense microstructure and higher homogeneity (Figure 9a).

Similar observations were made by Fonseca Coelho et al. [23] on the microstructure of gypsum-based composites containing sisal fibres. They revealed the presence of several pores as a result of the incomplete growth of calcium sulphate dihydrate crystals, which were themselves interlocked and, therefore, reduced the overall porosity. Porosity analysis showed that specimens made with sisal fibres had more pores (apparent porosity increases of 4%) due to the lack of interaction between the plant fibres and gypsum.

**Figure 10:**
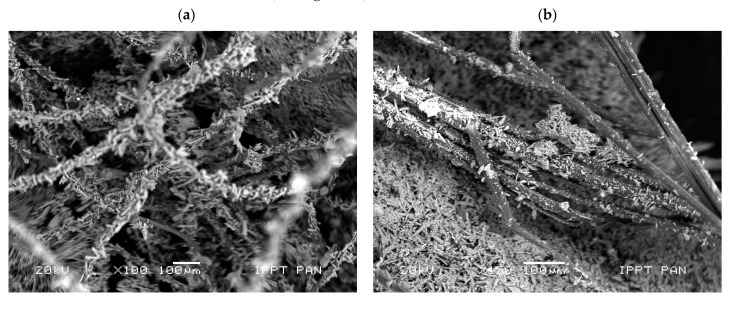
Microphotograph of the analysed fresh split surface of gypsum-based composites: (**a**) with sheep wool fibres and (**b**) with hemp fibres (scanning electron microscope, scale bar = 100 µm).

**Figure 11:**
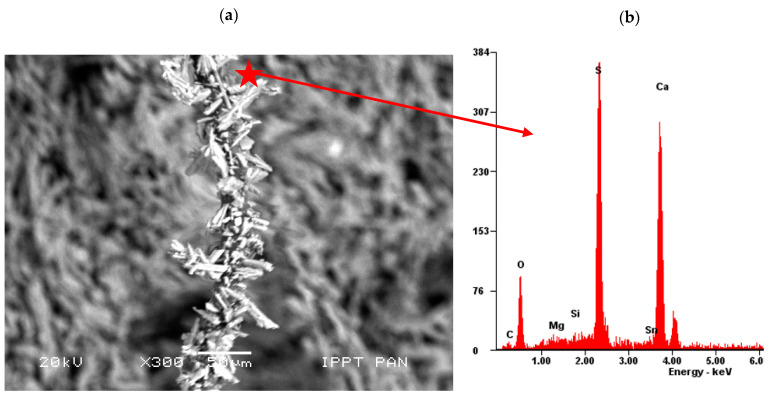
A single sheep wool fibre completely overgrown with gypsum crystals: (**a**) SEM microphotograph and (**b**) EDS analysis: S-Sulphur, Ca-Calcium, Si-Silicon, Mg-Magnesium, O-Oxygen (scanning electron microscope, scale bar = 50 µm).

As Figure 10 shows, in the investigated specimens, the sheep wool fibres were distributed over the entire surface (Figure 10a), whereas the hemp fibres were positioned in “nests” (Figure 10b). The morphological analysis of the fresh fractured surface of the gypsum-based composites confirmed very good adhesion between sheep wool fibres and the matrix (see Figure 11).

All the visible sheep wool fibres were very tightly covered with gypsum crystals (Figure 10a and Figure 11, whereas hemp fibres were only partially covered with gypsum crystals (Figure 10b). Due to the surface of the sheep wool fibres, it was not possible to perform a micro-area analysis on the clean fibre. On the contrary, EDS analysis revealed the same peaks as for the reference specimens without the addition of fibres (compare Figure 9 and Figure 11).

The delamination of hemp fibres that was observed on the constituent materials is also visible in Figure 10b, which was taken on the gypsum paste specimens. Such a phenomenon was also noticed in other studies on gypsum composites containing hemp fibres, despite the latter being biologically and chemically treated [16]. Finally, fibre delamination was also observed by Iucolano et al. [12], who investigated other plant origin fibres (i.e., abaca fibres) in gypsum containing bassanite (CaSO_4_·0.5H_2_O) and anhydrite (CaSO_4_), but without gypsum itself (CaSO_4_·2H_2_O).

The observed microstructure of gypsum paste reinforced with sheep wool or hemp fibres is consistent with the results of the mechanical tests, as shown in the previous section. In particular, A_F_ is higher in Series WF (wool reinforced) than in Series HF (hemp reinforced) because of the higher bond strength developed at the fibre–gypsum interface. Indeed, the gypsum matrix tends to corrugate the original smooth surface of the wool fibre of Figure 1a, on which several crystals are present after extruding it (see Figure 11). Such a phenomenon does not occur in hemp fibre, which tends to delaminate outside (Figure 1a) and inside the matrix (Figure 10b). Such delamination could be caused by shorter d-spacings for hemp (Figure 2b), which are three times lower than in wool. As a consequence, having a more densely packed structure, hemp is much stiffer than wool and tends to delaminate.

Finally, it is worth noting that to develop a higher mechanical bond with a cementitious matrix, industrial fibres are frequently modified by roughening the surface or by inducing mechanical deformations [24]. After modification, these fibres can appear as intended, crimped, or waved as on the surface of the sheep wool fibre with the gypsum crystals shown in Figure 11. The modified surface of wool fibres is naturally produced by the presence of gypsum. Therefore, it seems very effective to reinforce gypsum manufacts with sheep wool and not only because it is currently a special waste. Indeed, wool can perform better than other natural and industrial fibres to reduce the economic and environmental impact of the construction industry.

## 4. Conclusions

Based on the research carried out on gypsum composites, reinforced with hemp or sheep wool fibres, the following conclusions can be drawn:

- Sheep wool fibres are characterized by uniform dimensions, whereas hemp fibres show high dimensional variability and tend to delaminate.
- XRD analysis shows that hemp fibres are semi-crystalline, with a high crystallinity index (i.e., *Ic* = 84.5%). Sheep wool fibres also show a crystal type structure, but with a relatively low degree of crystallographic order.
- Mechanical tests reveal the fracture toughness depends on the type of bio-fibre.
- The fracture toughness of the wool-reinforced gypsum is remarkably larger than that measured on hemp-reinforced composites, due to a better adhesion of wool with the gypsum matrix.
- SEM analysis performed on fibre-reinforced gypsum showed a dense matrix and a high homogeneity of matrix with sheep wool, whereas the matrix with hemp fibres contained empty air-voids. This is due to the delamination of the hemp fibres.
- Very good adhesion of the sheep wool fibres with the gypsum matrix occurs. Indeed, gypsum modifies the surface of wool fibres, which appears completely covered by crystals. The same does not occur as much on the surface of hemp fibres.

As a large content of wool can also produce a deflection hardening behaviour of gypsum manufacts [24], future work will be devoted to tailor the wool reinforced gypsum composites containing more than 1% in volume of fibres.

## Figures and Tables

**Table 1:** The main mechanical properties measured in the mechanical tests.

Series	*η_p_*	st. dev.	*P_max_*	st. dev.	*A_F_*	st. dev.	*P_c_*	st. dev.
	(mm)		(N)		(mm)		(N)	
ref.	1.29	0.03	2330	138	0.004	0.001	22307	908
WF	1.15	0.16	1876	232	1.026	0.131	15652	3760
HF	1.21	0.07	1656	239	0.376	0.113	1739	2320

## Data Availability

The data presented in this study cannot be shared at this time as the data also forms part of an ongoing study.
